# Surrogate insulin resistance indices are associated with left atrial thrombus in patients with nonvalvular atrial fibrillation

**DOI:** 10.3389/fragi.2025.1571609

**Published:** 2025-09-03

**Authors:** You Zhou, Xuewen Song, Jifang Ma, Xianqing Wang, Haixia Fu

**Affiliations:** ^1^ Heart center of Henan Provincial People’s Hospital, Central China Fuwai Hospital, Central China Fuwai Hospital of Zhengzhou University, Zhengzhou, Henan, China; ^2^ Department of Hematology, The Affiliated Cancer Hospital of Zhengzhou University & Henan Cancer Hospital, Hemostasis and Thrombosis Diagnostic Engineering Research Center of Henan Province, Zhengzhou, China

**Keywords:** triglyceride glucose index, triglyceride glucose–body mass index, insulin resistance, left atrial thrombus, atrial fibrillation

## Abstract

**Introduction:**

Atrial fibrillation is the most common cardiac arrhythmia with increased risk of thromboembolic events. Surrogate insulin resistance indices, triglyceride glucose (TyG) index and triglyceride glucose-body mass index (TyG-BMI index), are predictors of adverse outcomes in patients with cardiovascular diseases. In this study, we aimed to determine the association between insulin resistance indices and left atrial thrombus (LAT) in patients with nonvalvular atrial fibrillation (NVAF).

**Methods:**

A total of 466 patients with documented NVAF who underwent transesophageal echocardiography were studied retrospectively. Demographic data, laboratory results, echocardiographic measurements and medication were collected. Logistic regression analysis was performed to determine the association between insulin resistance indices and risk of LAT. C-statistic was calculated to determine the incremental value of insulin resistance indices in predicting LAT compared with CHA2DS2-VASc score.

**Results:**

LAT were identified in 46 patients (9.87%). In the full adjustment model, elevated TyG index [per 1 standard deviation (SD) increment; odds ratio (OR): 1.588; 95% confidence interval (CI): 1.125-2.241, P = 0.009] and TyG-BMI index (per 1 SD increment; OR: 1.570; 95% CI: 1.142-2.160, P = 0.005) were significantly associated with high risk of LAT. Compared to the lowest quartile, ORs for the highest quartile were 3.691 (95% CI: 1.126-12.096, P = 0.031) for TyG index and 10.302 (95% CI: 2.232-47.556, P = 0.003) for TyG-BMI index. Compared with the CHA2DS2-VASc score alone, insulin resistance indices incorporated into the CHA2DS2-VASc score remain the predictive ability of LAT.

**Conclusion:**

The present study suggests that TyG index and TyG-BMI index are new predictors for LAT in patients with NVAF.

## 1 Introduction

Atrial fibrillation (AF) is the most common cardiac arrhythmia. The currently estimated prevalence of AF in adults is between 2% and 4%, which is expected to continue rising due to extended longevity (Hindricks et al., 2021). AF carries a significant worldwide health burden and is an important contributor to stroke, resulting in substantial morbidity and mortality. Nearly 30% of AF patients with strokes die within 1 year, and 15%–30% of stroke survivors suffer from permanent disability (Menke et al., 2010). Left atrial thrombus (LAT) formation is recognized as the primary cause of stroke in patients with AF. Therefore, it is essential to identify AF patients at high risk of LAT and provide appropriate therapy to protect them against stroke.

Cardiovascular risk factors and concomitant diseases are recognized as an integral part of the “Atrial fibrillation Better Care (ABC)” approach in AF management (Hindricks et al., 2021). The multifaceted dimensions of the ‘C’ include obesity, dyslipidemia, and glucose intolerance, which collectively form the framework of metabolic syndrome. Insulin resistance is the pivotal pathogenic component of metabolic syndrome and also contributes to the development of cardiovascular diseases in diabetic and nondiabetic subjects (Mancusi et al., 2019). Therefore, insulin resistance is a cause and a predictor of cardiovascular diseases in both the general populations and subjects with diabetes. Insulin resistance accelerates the progression of AF and atrial remodeling by systemic inflammation, altered energy supply, glucotoxicity, and lipotoxicity (Bode et al., 2024). These metabolic disturbances confer the additional risk of stroke for patients with AF (Bode et al., 2024; Hajhosseiny et al., 2015; Tsai et al., 2014). Cardiometabolic risk factors are modifiable, and strokes associated with AF are usually recurrent and severe; thus, finding a convenient and reliable indicator to detect insulin resistance and predict thromboembolic risk in patients with AF is of particular importance.

The triglyceride-glucose (TyG) index and the triglyceride-glucose–body mass index (TyG–BMI) are easily accessible surrogate markers of insulin resistance. Several studies have provided robust evidence that these indices are associated with the development and prognosis of cardiovascular disease, including AF (Lo et al., 2023; Cui et al., 2024; Azarboo et al., 2024). However, the predictive value of the two surrogate insulin resistance indices for LAT in patients with nonvalvular atrial fibrillation (NVAF) remains unknown. Accordingly, we conducted this study to investigate the association between insulin resistance indices and LAT in patients with NVAF who are undergoing transesophageal echocardiography (TEE).

## 2 Methods

### 2.1 Study population

**FIGURE 1:**
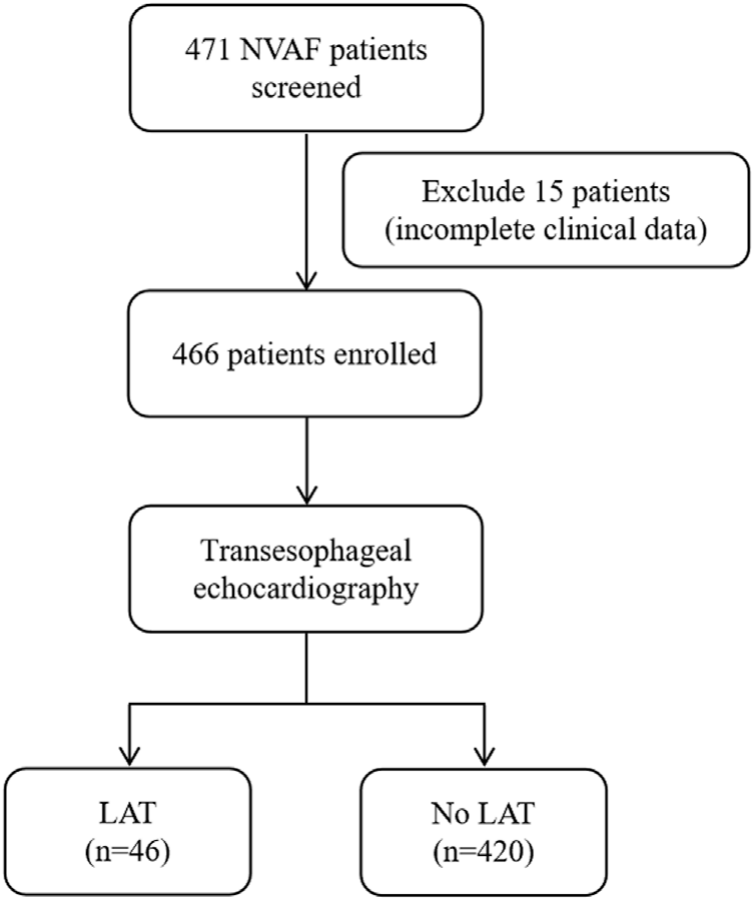
Flow diagram showing screening and recruitment of the study population. NVAF, nonvalvular atrial fibrillation; LAT, left atrial thrombus.

Consecutive hospitalized patients with NVAF who underwent TEE to determine LAT in Henan Provincial People’s Hospital and Fuwai Central China Cardiovascular Hospital between January 2015 and September 2022 were retrospectively enrolled. NVAF was defined according to the guideline as AF patients without moderate or severe mitral stenosis and mechanical prosthetic heart valve(s) (Hindricks et al., 2021). First-diagnosed AF was defined as AF not diagnosed previously, irrespective of its duration or the presence/severity of AF-related symptoms (Hindricks et al., 2021). Patients with incomplete data were excluded. The flowchart of patient inclusion is shown in Figure 1.

### 2.2 Data collection

Patients’ clinical data prior to TEE were retrieved from medical records, including age, gender, type of AF, comorbidities (coronary heart disease, heart failure, hypertension, diabetes, and history of stroke), oral anti-coagulant therapy, laboratory variables, and echocardiographic parameters. Venous blood samples after 12-h fasting were collected from each patient after admission and before TEE examination. All biochemical tests were carried out in the laboratory of the testing center for examination by using standard techniques. The estimated glomerular filtration rate was calculated using the Cockcroft–Gault equation. Body mass index (BMI) was calculated as weight divided by height squared (kg/m^2^). The CHA_2_DS_2_-VASc score was calculated from the sum of the risk factors of congestive heart failure, hypertension, age ≥ 75 years, diabetes mellitus, stroke, vascular disease, age 65 years–74 years, and sex (female); weighing each by 1, except for stroke and age ≥ 75 years, which were weighed by 2 (Hindricks et al., 2021). In the CHA_2_DS_2_-VASc scores, a score of ≥ 2 in men or ≥ 3 in women was categorized as high risk (Hindricks et al., 2021)

### 2.3 Equations of insulin resistance indices

Insulin resistance indices were calculated using the following equations: (Er et al., 2016)

$$\text{TyG index}=\mathrm{Ln}\;\left[\text{fasting triglyceride }\left(\text{mg}/\text{dL}\right)\times \text{fasting glucose }\left(\text{mg}/\text{dL}\right)/2\right].$$

Tyg−BMI index=TyG index×BMI.

### 2.4 Echocardiographic parameters

After admission, transthoracic echocardiography and TEE were performed according to the international guidelines. LAT referred to a highly reflective and well-circumscribed mass with uniform consistency and different texture from that of the atrial wall in TEE (Hahn et al., 2013). The left atrial diameter and left ventricular ejection fraction (LVEF) were measured from the M-mode or 2D view in the parasternal long-axis view (Hahn et al., 2013).

### 2.5 Statistical analysis

Continuous variables were presented as mean ± standard deviation (SD) and compared by Students’ t-tests. Categorical variables in each group were presented as numbers and percentages and compared by using the chi-square test. Spearman’s test was utilized for describing the correlation between insulin resistance indices and clinical variables. Restricted cubic splines were plotted to illustrate the dose–response association between insulin resistance indices and the risk of LAT. Four knots were placed at the 5th, 35th, 65th, and 95th percentiles of the insulin resistance indices. The univariate logistic regression model was used to assess the association between the indices and LAT, followed by multivariate adjustments. Model 1 was also adjusted for age and gender. Model 2 was additionally adjusted for clinical thrombotic factors (coronary heart diseases, heart failure, hypertension, diabetes, and stroke). Model 3 was adjusted for factors in model 2 and further adjusted for paroxysmal atrial fibrillation, oral anticoagulant, left atrial diameter, and LVEF. The odds ratio (OR) and 95% confidence interval (CI) were calculated to show the risk. C-statistic was calculated to determine the incremental value of insulin resistance indices in predicting LAT compared with the original CHA_2_DS_2_-VASc score. C-statistic was compared by using the DeLong test. All significance tests were two-tailed, and p-values < 0.05 were considered statistically significant. SPSS Statistics 26.0 (SPSS, Chicago, IL, United States) and R 4.4.0 (R Core Team, Vienna, Austria) were used to perform the statistical analysis.

## 3 Results

### 3.1 Patient characteristics

**FIGURE 2:**
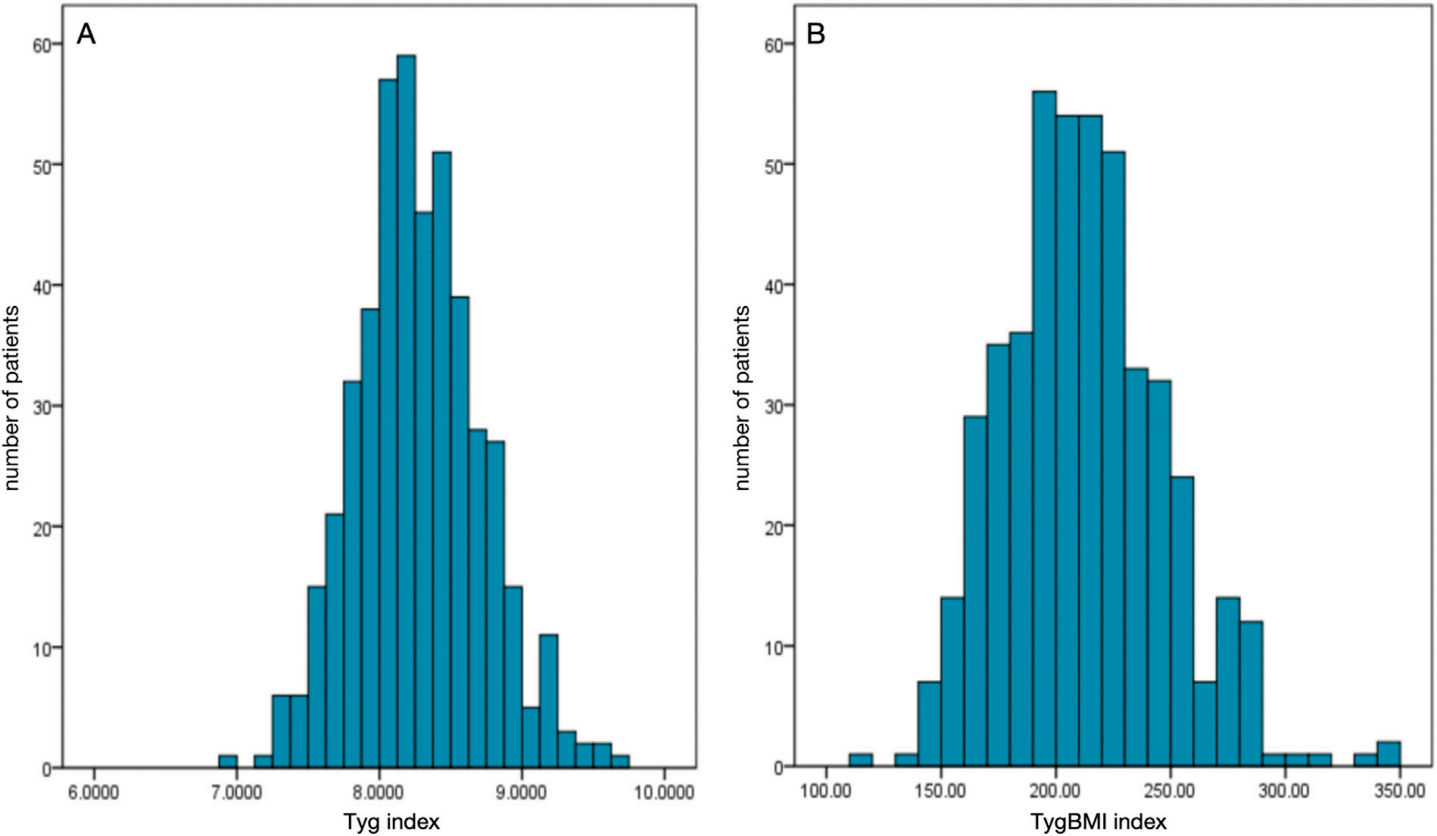
Distributions of the insulin resistance indices among the enrolled patients. **(A)** Distribution of the triglyceride-glucose index (TyG index). **(B)** Distribution of the triglyceride-glucose–body mass index (TyG–BMI).

A total of 466 patients were enrolled in this study. The average age of the included patients was 61.16 ± 9.95 years, and 66.5% of them were men; 61.2% of participants were first diagnosed with NVAF at admission, and 27.3% of these included patients received oral anticoagulants at admission. The average TyG index and TyG–BMI were 8.27 ± 0.44 and 212.19 ± 35.02, respectively. LAT was found in 46 patients (9.87%). Patients with LAT were more likely to have heart failure, with higher BMI, lower LVEF, and higher levels of triglyceride than those without LAT. Both the TyG index and TyG–BMI were significantly higher in patients with LAT (Table 1). The distributions of the insulin resistance indices are shown in Figure 2.

**TABLE 1:** Baseline characteristics of the participants stratified by left atrial thrombus.

Variable	Total (n = 466)	No LAT (n = 420)	LAT (n = 46)	P-value
Male, n (%)	310 (66.5%)	277 (66.0%)	33 (71.7%)	0.430
Age (years)	61.16 ± 9.95	61.38 ± 9.72	59.13 ± 11.80	0.145
Body mass index (kg/m^2^)	25.61 ± 3.75	25.46 ± 3.76	26.83 ± 2.90	0.018
Paroxysmal atrial fibrillation, n (%)	110 (23.6%)	99 (23.6%)	11 (23.9%)	0.959
First-diagnosed atrial fibrillation, n (%)	285 (61.2%)	258 (61.4%)	27 (58.7%)	0.718
Coronary heart diseases, n (%)	84 (18.0%)	79 (18.8%)	5 (10.9%)	0.184
Heart failure, n (%)	133 (28.5%)	114 (27.1%)	19 (41.3%)	0.043
Hypertension, n (%)	193 (41.4%)	178 (42.4%)	15 (32.6%)	0.201
Diabetes, n (%)	66 (14.2%)	60 (14.3%)	6 (13.0%)	0.819
Stroke, n (%)	67 (14.4%)	58 (13.8%)	9 (19.6%)	0.291
Oral anticoagulant, n (%)	127 (27.3%)	112 (26.7%)	15 (32.6%)	0.390
Beta-blockers, n (%)	135 (29.0%)	121 (28.8%)	14 (30.4%)	0.818
Renin–angiotensin system inhibitors, n (%)	73 (15.7%)	64 (15.2%)	9 (19.5%)	0.443
Spironolactone, n (%)	63 (13.5%)	58 (13.8%)	5 (10.9%)	0.580
Statin, n (%)	95 (20.4%)	88 (21.0%)	7 (15.2%)	0.359
Left atrium diameter (mm)	49.28 ± 9.28	49.29 ± 9.44	49.22 ± 7.60	0.957
Left ventricular ejection fraction (%)	56.67 ± 9.04	57.08 ± 8.61	55.89 ± 7.80	0.025
eGFR (mL/min/1.73 m^2^)	85.35 ± 20.39	85.03 ± 20.22	88.31 ± 21.94	0.301
Triglyceride (mmol·L^-1^)	1.09 ± 0.39	1.07 ± 0.38	1.24 ± 0.46	0.006
Total cholesterol (mmol/L)	3.67 ± 0.88	3.65 ± 0.88	3.91 ± 0.85	0.061
HDL-C (mmol/L)	1.13 ± 0.30	1.13 ± 0.30	1.08 ± 0.25	0.223
LDL-C (mmol/L)	2.24 ± 0.76	2.22 ± 0.76	2.45 ± 0.75	0.056
Fasting plasma glucose (mmol/L)	4.97 ± 1.36	4.95 ± 1.35	5.11 ± 1.46	0.452
Glycated hemoglobin (%)	6.00 ± 0.91	5.99 ± 0.92	6.06 ± 0.83	0.595
CHA_2_DS_2_-VASc score	2.13 ± 1.53	2.13 ± 1.50	2.07 ± 1.73	0.774
TyG index	8.27 ± 0.44	8.26 ± 0.43	8.45 ± 0.44	0.004
TyG–BMI	212.19 ± 35.02	210.61 ± 35.50	226.62 ± 26.46	0.003

Abbreviations: LAT, left atrial thrombus; BMI, body mass index; eGFR, estimated glomerular filtration rate; HDL-C, high-density lipoprotein cholesterol; LDL-C, low-density lipoprotein cholesterol; TyG index, triglyceride-glucose index.

The medians (interquartile ranges) of the TyG index and TyG–BMI were 8.25 (7.99, 8.54) and 209.97 (186.35, 234.21), respectively. Table 2, Table 3 demonstrate the baseline characteristics of patients according to the baseline TyG index quartiles and TyG–BMI quartiles, respectively. Participants with higher quartiles of TyG index or TyG–BMI were more likely to have higher BMI, higher proportion of history of diabetes, lower LVEF, higher levels of triglycerides, low-density lipoprotein cholesterol (LDL-C), fasting plasma glucose, glycated hemoglobin, and lower level of high-density lipoprotein cholesterol (HDL-C). Patients with higher quartiles of TyG–BMI were also more likely to have a higher history of hypertension.

**TABLE 2:** Baseline characteristics of the participants by the quartiles of the TyG index.

Variable	Quartile 1 (n = 116)	Quartile 2 (n = 117)	Quartile 3 (n = 117)	Quartile 4 (n = 116)	P-value
Male, n (%)	76 (65.5%)	75 (64.1%)	78 (66.7%)	81 (69.8%)	0.818
Age (years)	62.28 ± 11.13	61.31 ± 9.40	60.44 ± 9.58	60.62 ± 9.62	0.489
Body mass index (kg/m^2^)	23.91 ± 3.31	25.77 ± 3.66	26.32 ± 3.91	26.37 ± 3.38	<0.001
Paroxysmal atrial fibrillation, n (%)	26 (22.4%)	29 (24.8%)	29 (24.8%)	26 (22.4%)	0.948
First-diagnosed atrial fibrillation, n (%)	62 (53.4%)	76 (65.0%)	75 (64.1%)	72 (62.1%)	0.253
Coronary heart diseases, n (%)	16 (13.8%)	21 (17.9%)	24 (20.5%)	23 (19.8%)	0.542
Heart failure, n (%)	34 (29.3%)	32 (27.4%)	30 (25.6%)	37 (31.9%)	0.744
Hypertension, n (%)	48 (41.4%)	52 (44.4%)	44 (37.6%)	49 (42.4%)	0.759
Diabetes, n (%)	6 (5.2%)	15 (12.8%)	13 (11.1%)	32 (27.6%)	<0.001
Stroke, n (%)	14 (12.1%)	18 (15.4%)	19 (16.2%)	18 (15.5%)	0.812
Oral anticoagulant, n (%)	36 (31.0%)	31 (26.5%)	28 (23.9%)	32 (27.6%)	0.676
Beta-blockers, n (%)	24 (20.7%)	45 (38.5%)	34 (29.1%)	32 (27.6%)	0.028
Renin–angiotensin system inhibitors, n (%)	14 (12.1%)	24 (20.5%)	16 (13.7%)	19 (16.4%)	0.307
Spironolactone, n (%)	15 (12.9%)	16 (13.7%)	12 (10.3%)	20 (17.2%)	0.480
Statin, n (%)	26 (22.4%)	25 (21.4%)	22 (18.8%)	22 (19.0%)	0.876
Left atrium diameter (mm)	50.92 ± 9.93	48.97 ± 8.86	47.74 ± 8.99	49.46 ± 9.10	0.081
Left ventricular ejection fraction (%)	58.89 ± 7.46	57.04 ± 9.48	56.75 ± 8.82	54.07 ± 9.67	0.001
eGFR (mL/min/1.73 m^2^)	85.34 ± 21.55	85.24 ± 17.31	87.88 ± 21.04	82.95 ± 21.36	0.336
Triglyceride (mmol·L^-1^)	0.71 ± 0.13	0.94 ± 0.14	1.14 ± 0.20	1.56 ± 0.40	<0.001
Total cholesterol (mmol/L)	3.31 ± 0.76	3.59 ± 0.80	3.74 ± 0.87	4.06 ± 0.93	<0.001
HDL-C (mmol/L)	1.22 ± 0.33	1.17 ± 0.31	1.09 ± 0.30	1.04 ± 0.23	<0.001
LDL-C (mmol/L)	1.93 ± 0.63	2.18 ± 0.73	2.31 ± 0.73	2.55 ± 0.82	<0.001
Fasting plasma glucose (mmol/L)	4.23 ± 0.63	4.61 ± 0.70	5.00 ± 0.86	6.03 ± 2.01	<0.001
Glycated hemoglobin (%)	5.69 ± 0.50	5.86 ± 0.68	5.99 ± 0.89	6.44 ± 1.23	<0.001
CHA_2_DS_2_-VASc score	2.08 ± 1.33	2.16 ± 1.65	1.99 ± 1.49	2.28 ± 1.62	0.531

Abbreviations: eGFR, estimated glomerular filtration rate; HDL-C, high-density lipoprotein cholesterol; LDL-C, low-density lipoprotein cholesterol; TyG index, triglyceride-glucose index.

**TABLE 3:** Baseline characteristics of the participants by the quartiles of the TyG–BMI.

Variable	Quartile 1 (n = 116)	Quartile 2 (n = 117)	Quartile 3 (n = 117)	Quartile 4 (n = 116)	P-value
Male, n (%)	69 (59.5%)	81 (69.2%)	78 (66.7%)	82 (70.7%)	0.276
Age (years)	60.83 ± 10.85	61.90 ± 9.53	61.85 ± 10.68	60.05 ± 8.58	0.428
Body mass index (kg/m^2^)	21.39 ± 1.60	24.24 ± 1.24	26.57 ± 1.36	30.19 ± 2.74	<0.001
Paroxysmal atrial fibrillation, n (%)	29 (25.0%)	25 (21.4%)	26 (22.2%)	30 (25.9%)	0.825
First-diagnosed atrial fibrillation, n (%)	69 (59.5%)	78 (66.7%)	62 (53.0%)	76 (65.5%)	0.119
Coronary heart diseases, n (%)	12 (10.3%)	25 (21.4%)	23 (19.7%)	24 (20.7%)	0.099
Heart failure, n (%)	37 (31.9%)	34 (29.1%)	30 (25.6%)	32 (27.6%)	0.755
Hypertension, n (%)	39 (33.6%)	43 (36.8%)	51 (43.6%)	60 (51.7%)	0.026
Diabetes, n (%)	7 (6.0%)	17 (14.5%)	17 (14.5%)	25 (21.6%)	0.009
Stroke, n (%)	18 (15.5%)	19 (16.2%)	17 (14.5%)	15 (12.9%)	0.904
Oral anticoagulant, n (%)	31 (26.7%)	23 (19.7%)	39 (33.3%)	34 (29.3%)	0.119
Beta-blockers, n (%)	28 (24.1%)	28 (23.9%)	36 (30.8%)	43 (37.1%)	0.084
Renin–angiotensin system inhibitors, n (%)	18 (15.5%)	17 (14.5%)	17 (14.5%)	21 (18.1%)	0.861
Spironolactone, n (%)	14 (12.1%)	16 (13.7%)	16 (10.3%)	17 (14.7%)	0.952
Statin, n (%)	18 (15.5%)	22 (18.8%)	26 (22.2%)	29 (15.0%)	0.303
Left atrium diameter (mm)	48.65 ± 10.21	49.15 ± 8.86	50.41 ± 9.91	48.91 ± 8.03	0.502
Left ventricular ejection fraction (%)	58.00 ± 9.04	57.40 ± 7.48	56.61 ± 9.52	54.70 ± 9.72	0.037
eGFR (mL/min/1.73 m^2^)	88.16 ± 20.64	85.15 ± 21.05	83.48 ± 21.25	84.62 ± 18.48	0.343
Triglyceride (mmol·L^-1^)	0.87 ± 0.29	1.06 ± 0.35	1.11 ± 0.37	1.31 ± 0.44	<0.001
Total cholesterol (mmol/L)	3.68 ± 0.85	3.58 ± 0.84	3.63 ± 0.92	3.81 ± 0.91	0.232
HDL-C (mmol/L)	1.26 ± 0.38	1.14 ± 0.24	1.09 ± 0.28	1.03 ± 0.23	<0.001
LDL-C (mmol/L)	2.19 ± 0.71	2.12 ± 0.73	2.26 ± 0.82	2.40 ± 0.77	0.045
Fasting plasma glucose (mmol/L)	4.34 ± 0.71	4.90 ± 1.60	5.07 ± 1.02	5.57 ± 1.60	<0.001
Glycated hemoglobin (%)	5.72 ± 0.53	5.97 ± 1.04	5.97 ± 0.72	6.33 ± 1.12	<0.001
CHA_2_DS_2_-VASc score	2.03 ± 1.54	2.15 ± 1.55	2.20 ± 1.67	2.13 ± 1.35	0.853

Abbreviations: eGFR, estimated glomerular filtration rate; HDL-C, high-density lipoprotein cholesterol; LDL-C, low-density lipoprotein cholesterol; TyG–BMI, triglyceride-glucose–body mass index.

### 3.2 Correlation between insulin resistance indices and baseline variables

Correlation analysis revealed significant but generally weak associations (Table 4). Both the TyG index and TyG–BMI showed positive correlations with LDL-C (TyG: r = 0.285, p < 0.001; TyG–BMI: r = 0.132, p = 0.004) and glycated hemoglobin (TyG: r = 0.346, p < 0.001; TyG–BMI: r = 0.214, p < 0.001). Conversely, negative correlations were observed for both the indices with LVEF (TyG: r = −0.179, p < 0.001; TyG–BMI: r = −0.120, p = 0.012) and HDL-C (TyG: r = −0.259, p < 0.001; TyG-BMI: r = −0.270, p < 0.001). Notably, only the TyG index exhibited a significant positive correlation with total cholesterol (r = 0.306, p < 0.001).

**TABLE 4:** Correlation analysis between insulin resistance indices and baseline variables.

Variable	TyG index		TyG–BMI	
	Correlation coefficient	P-value	Correlation coefficient	P-value
Age	−0.088	0.057	−0.079	0.087
Left atrium diameter	−0.033	0.490	0.031	0.520
Left ventricular ejection fraction	−0.179	<0.001	−0.120	0.012
eGFR	−0.065	0.160	−0.040	0.388
Total cholesterol	0.306	<0.001	0.090	0.052
HDL-C	−0.259	<0.001	−0.270	<0.001
LDL-C	0.285	<0.001	0.132	0.004
Glycated hemoglobin	0.346	<0.001	0.214	<0.001
CHA_2_DS_2_-VASc score	0.024	0.611	0.006	0.894

Abbreviations: TyG index, triglyceride-glucose index; BMI, body mass index; eGFR, estimated glomerular filtration rate; HDL-C, high-density lipoprotein cholesterol; LDL-C, low-density lipoprotein cholesterol.

### 3.3 Dose–response relationship between insulin resistance indices and risk of LAT

**FIGURE 3:**
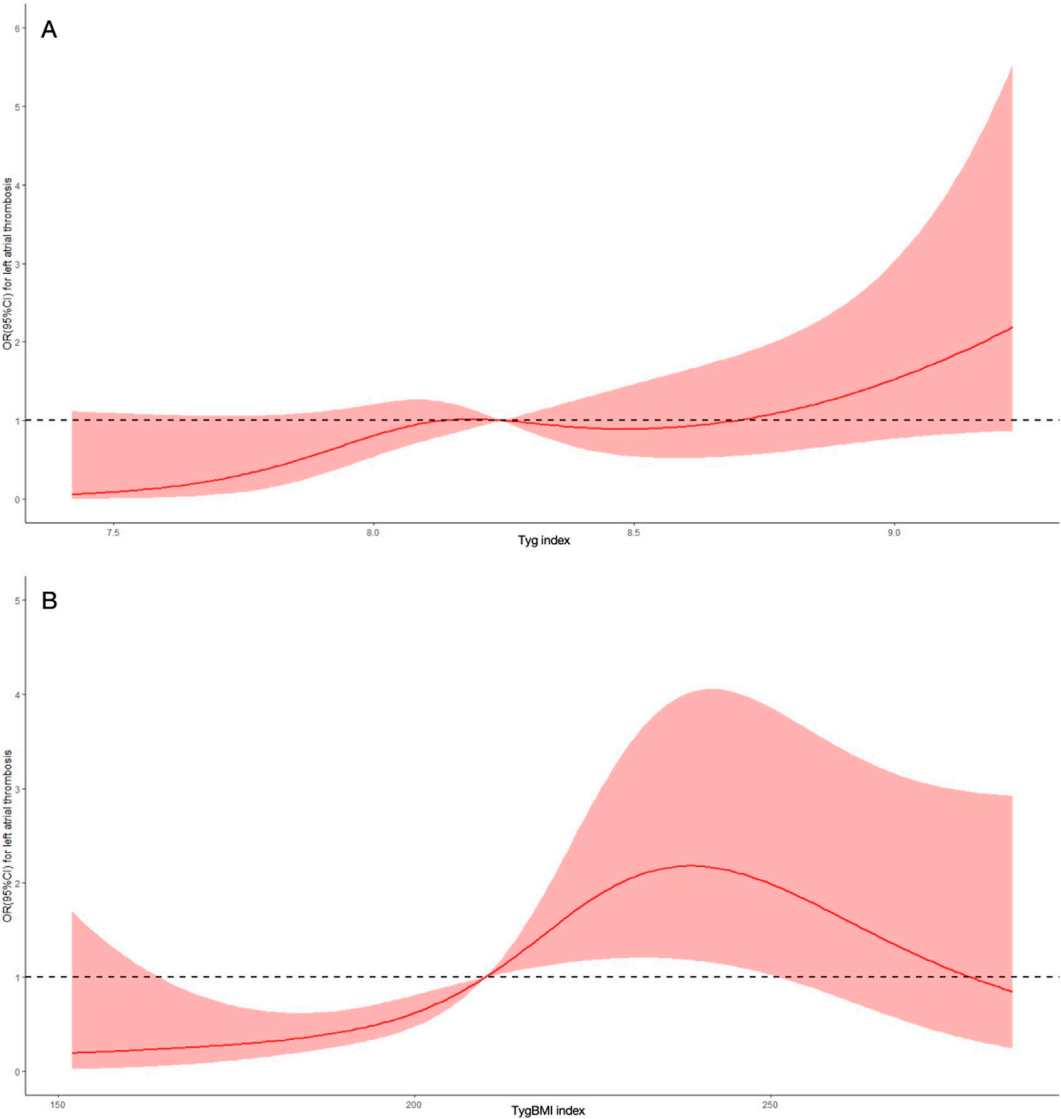
Dose-response curve of insulin resistance indices and the risk of left atrial thrombus. **(A)** Dose-response curve of the triglyceride glucose index (TyG index). **(B)** Dose-response curve of the triglyceride glucose-body mass index (TyG-BMI index). OR, odds ratio; CI, confidence interval.

To continuously assess the association of insulin resistance indices with the risk of LAT, dose–response curves were constructed (Figure 3). Both the TyG index (p for non-linearity = 0.002) and TyG–BMI (p for non-linearity <0.001) had a nonlinear and positive correlation with the risk of LAT.

### 3.4 Logistic regression analysis of the association between insulin resistance indices and LAT

The regression analysis indicated that the risk of LAT increased more than 50% per SD after multivariate adjustment by using the SD-transformed TyG index (OR 1.588, 95% CI: 1.125–2.241) and TyG–BMI (OR 1.570, 95% CI: 1.142–2.160) as continuous variables. After adjustment for potential confounding factors, compared with the lowest quartile of the TyG index, the risk of LAT in participants with the highest quartile was significantly higher (OR 3.691, 95% CI: 1.126–12.096). In comparison to the first quartile of TyG–BMI, a higher risk of LAT was observed in patients in the third (OR 11.547, 95% CI: 2.509–53.146) and the fourth quartiles (OR 10.302, 95% CI: 2.232–47.556) in model 3 (Table 5).

**TABLE 5:** Logistics regression analysis to examine the association between insulin resistance indices and LAT.

Variable	Unadjusted model		Model 1		Model 2		Model 3	
	Or (95% CI)	P-value	Or (95% CI)	P-value	Or (95% CI)	P-value	Or (95% CI)	P-value
TyG index (per SD)	1.544 (1.143–2.086)	0.005	1.543 (1.137–2.093)	0.005	1.617 (1.167–2.240)	0.004	1.588 (1.125–2.241)	0.009
TyG index (quartiles)								
Quartile 1	Reference		Reference		Reference		Reference	
Quartile 2	3.806 (1.214–11.935)	0.022	3.781 (1.204–11.879)	0.023	4.032 (1.273–12.767)	0.018	3.058 (0.922–10.139)	0.068
Quartile 3	3.500 (1.106–11.076)	0.033	3.394 (1.070–10.762)	0.038	3.610 (1.130–11.540)	0.030	3.042 (0.915–10.112)	0.069
Quartile 4	4.158 (1.336–12.941)	0.014	4.015 (1.287–12.527)	0.017	4.293 (1.345–13.696)	0.014	3.691 (1.126–12.096)	0.031
P for trend		0.028		0.035		0.029		0.049
TyG–BMI (per SD)	1.536 (1.150–2.053)	0.004	1.501 (1.123–2.007)	0.006	1.613 (1.197–2.173)	0.002	1.570 (1.142–2.160)	0.005
TyG–BMI index (quartiles)								
Quartile 1	Reference		Reference		Reference		Reference	
Quartile 2	2.397 (0.604–9.506)	0.214	2.444 (0.613–9.734)	0.205	2.871 (0.709–11.620)	0.139	4.593 (0.912–23.138)	0.065
Quartile 3	7.303 (2.098–25.421)	0.002	7.487 (2.142–26.175)	0.002	9.338 (2.621–33.270)	0.001	11.547 (2.509–53.146)	0.002
Quartile 4	6.468 (1.841–22.727)	0.004	6.355 (1.801–22.415)	0.004	8.414 (2.319–30.527)	0.001	10.302 (2.232–47.556)	0.003
P for trend		<0.001		<0.001		<0.001		<0.001

Model 1: Adjusted for age and gender.

Model 2: Additionally adjusted for clinical thrombosis factors (coronary heart diseases, heart failure, hypertension, diabetes, and stroke).

Model 3: Further adjusted for paroxysmal atrial fibrillation, oral anticoagulant, left atrial diameter, and left ventricular ejection fraction.

Abbreviations: LAT, left atrial thrombus; OR, odds ratio; CI, confidence interval; TyG index, triglyceride–glucose index; BMI, body mass index; SD, standard deviation.

### 3.5 Subgroup analysis

**FIGURE 4:**
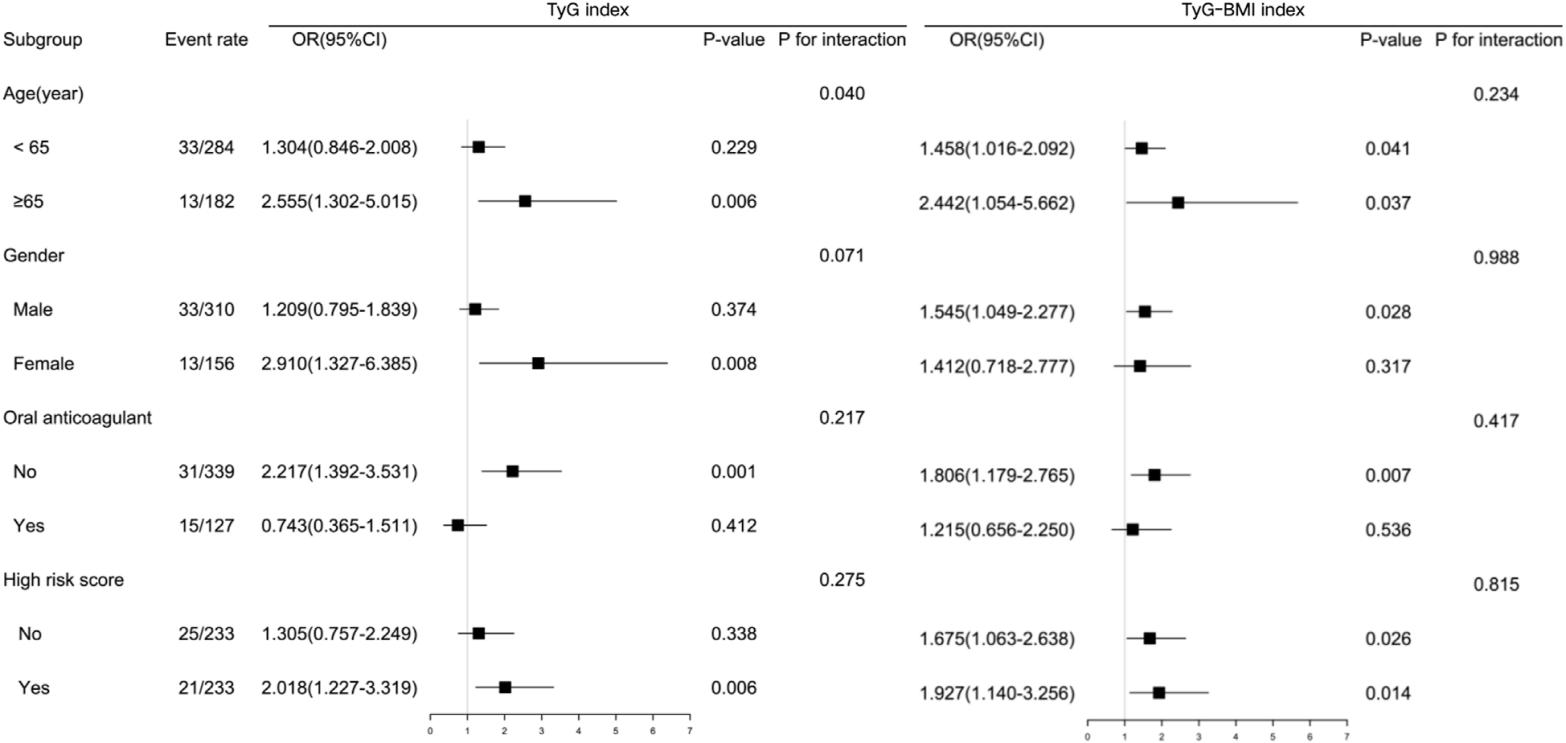
Subgroup-specific associations between insulin resistance indices and left atrial thrombus risk. Forest plot shows odds ratios (ORs) and 95% confidence intervals (CIs) per 1-SD increase in the triglyceride-glucose index (TyG index) or triglyceride-glucose–body mass index (TyG–BMI).

The subgroup analyses (Figure 4) demonstrated the predictive significance of the TyG index in specific groups. The specific groups were individuals aged < 65 years old, women, those receiving no anticoagulant at admission, and individuals without high CHA_2_DS_2_-VASc scores. The TyG–BMI maintained a significant association with LAT in both aged subgroups, men, individuals receiving no anticoagulant at admission, and both CHA_2_DS_2_-VASc score subgroups. There existed an interaction in the subgroup of age regarding the association between the TyG index and risk of LAT.

### 3.6 Improvement for risk prediction for LAT by CHA_2_DS_2_-VASc score

In patients receiving no anticoagulant at admission, despite the inability to predict LAT by CHA_2_DS_2_-VASc score (C-statistic: 0.527, 95% CI: 0.404–0.650, p = 0.746) alone, the TyG index incorporated into the CHA_2_DS_2_-VASc score (C-statistic: 0.659, 95% CI: 0.567–0.751, p = 0.003) and TyG–BMI incorporated into the CHA_2_DS_2_-VASc score (C-statistic: 0.672, 95% CI: 0.581–0.762, p = 0.002) were still associated with risk of LAT. However, the difference in the predictive ability between the two composite schemes and the CHA_2_DS_2_-VASc score alone did not reach statistical significance (p = 0.109 and 0.098, respectively; Table 6).

**TABLE 6:** Discrimination ability of different indices.

Variable	C-statistic (95% CI)	P-value	ΔC-statistic (95% CI)	P-value
CHA_2_DS_2_-VASc score	0.527 (0.404,0.650)	0.622	reference	
TyG index	0.664 (0.576,0.753)	0.003	0.137 (-0.003,0.278)	0.055
TyG–BMI	0.678 (0.590,0.766)	0.001	0.151 (-0.013,0.315)	0.071
TyG index + CHA_2_DS_2_-VASc score	0.659 (0.567,0.751)	0.003	0.132 (-0.030,0.294)	0.109
TyG–BMI + CHA_2_DSc_2_-VASc score	0.672 (0.581,0.762)	0.002	0.145 (-0.027,0.317)	0.098

Abbreviations: CI, confidence interval; TyG index, triglyceride-glucose index; BMI, body mass index.

## 4 Discussion

Our principal finding was that elevated insulin resistance indices (TyG index and TyG–BMI) were associated with increased risk for LAT. Adding either index to the CHA_2_DS_2_-VASc score had the ability to predict LAT, but it did not have any significant improvement compared to the CHA_2_DS_2_-VASc score alone. To our knowledge, the present study is the first to examine the predictive value of insulin resistance for LAT in patients with NVAF.

Hitherto, there was a paucity of evidence regarding the association between insulin resistance or metabolic syndrome and LAT or stroke in patients with AF. Tsai CT et al. identified a graded association between the increasing number of components of metabolic syndrome and the risk of stroke in 721 patients with NVAF (Tsai et al., 2014). A study including 294 patients with NVAF demonstrated that metabolic syndrome was a strong risk factor for LAT (OR = 14.698, p < 0.001) (Chen et al., 2016). Insulin resistance is associated with various aspects of atrial remodeling, including increased oxidative stress, chronic inflammation, and interstitial fibrosis (Chan et al., 2019; Karam et al., 2017). Insulin resistance makes platelets insensitive to the anti-aggregating actions of prostaglandin I2 and nitric oxide and can increase adhesion-induced and thromboxane A2-dependent tissue factor expression in platelets (Gerrits et al., 2010). Insulin resistance will induce excessive glycosylation, which can promote endothelial injury, collagen crosslinking, and collagen deposition (Hill et al., 2021). These underlying mechanisms may contribute to thrombogenicity in patients with AF.

Previous research studies revealed that the TyG index and TyG–BMI were not only valid indicators for assessing insulin resistance but were also associated with the prognosis of cardiovascular diseases (Mancusi et al., 2019; Er et al., 2016). Euglycemic insulin clamp and intravenous glucose tolerance testing, the gold standards for insulin resistance testing, are invasive and expensive. These simple, convenient, and low-cost surrogates do not require insulin quantification and may be used in all subjects regardless of their insulin treatment status. A study involving 99 individuals with various degrees of body weight and glucose tolerance demonstrated that the TyG index was an optimal tool for the assessment of insulin resistance, with high sensitivity (96.5%) and specificity (85.0%) compared to the euglycemic–hyperinsulinemia clamp test (Guerrero-Romero et al., 2010). In the general population, the TyG index and TyG–BMI were significantly and positively associated with the risk of stroke (Shao et al., 2024; Jiang et al., 2024). Similar to these studies, our research confirmed the predictive value of insulin resistance indices for LAT formation in an NVAF population. Compared to the group without LAT, patients with LAT were predisposed to risk of cardiometabolic disorders, including higher BMI and triglycerides. The insulin resistance indices were positively correlated with LDL-C and glycated hemoglobin and negatively correlated with HDL-C. Patients with higher insulin resistance indices were more likely to suffer from diabetes. Thus, metabolic disturbances might be associated with higher risk of LAT, and the TyG index and TyG–BMI were indicators of cardiometabolic disorders. This study implied that insulin resistance would increase risk of LAT in patients with NVAF and that the TyG index and TyG–BMI were predictive indicators of LAT. While TyG indices showed statistically significant correlations with LAT risk, their modest effect sizes reflect the multifactorial nature of thrombogenesis—where metabolic drivers operate alongside hemodynamic, inflammatory, and pro-thrombotic factors. This precludes their standalone diagnostic use but supports their role as metabolic risk enhancers in integrated models. In subgroup analysis, the TyG–BMI was still associated with higher risk in groups aged <65, men, and those with a lower CHA_2_DS_2_-VASc score, while the TyG index did not reach significance. Nevertheless, several cohort studies showed that BMI was related to higher incidence of stroke in male and younger populations (Park et al., 2008; Gu et al., 2019; Cui et al., 2021). TyG–BMI might have unique characteristics compared to the TyG index.

The CHA_2_DS_2_-VASc score has been extensively validated and adopted in guidelines to stratify stroke risk (Hindricks et al., 2021). However, this score based on clinical factors has only a modest predictive value for identifying patients with high stroke risk. A prior study showed that the CHA_2_DS_2_-VASc score was of limited value for predicting LAT in patients with NVAF (Zhan et al., 2018). This questioned the CHA_2_DS_2_-VASc score’s ability in predicting strokes, mainly through the mechanism of cardiogenic embolism. Therefore, a scheme incorporating biomarkers may better predict LAT as a surrogate for cardioembolic risk. Adding insulin resistance indices to the CHA_2_DS_2_-VASc score provided the ability to predict LAT. The score based on clinical risk factors is a mere simplification of a more complex scenario. Some critical factors in thrombogenesis, such as insulin resistance, oxidative stress, and inflammation, are not reflected in the CHA_2_DS_2_-VASc score. Thus, risk stratification schemes including biomarkers, such as the TyG index and TyG–BMI, may offer improvements over the CHA_2_DS_2_-VASc score.

The observed association between elevated insulin resistance indices and LAT risk underscores the potential role of metabolic dysregulation in thrombogenesis. Therapeutic intensification targeting hyperglycemia or hypertriglyceridemia may attenuate LAT risk through multifaceted pathways. Improved glycemic control (e.g., via SGLT2 inhibitors or GLP-1 receptor agonists) may reduce atrial structural remodeling by mitigating inflammation, oxidative stress, and endothelial dysfunction—key drivers of the thrombogenic atrial substrate (Zhong et al., 2024; Lui et al., 2023; O'Keefe et al., 2021). Similarly, triglyceride-lowering agents (e.g., fibrates and omega-3 fatty acids) could diminish lipotoxicity-induced atrial fibrosis and dysregulated coagulation by suppressing pro-thrombotic adipokine release and restoring lipid metabolism homeostasis (O'Keefe et al., 2021; Reiner et al., 2024). Notably, these therapies may also indirectly reduce atrial stasis by improving cardiac diastolic function, particularly in comorbid metabolic syndrome or diabetes. Adjunctive metabolic optimization might synergistically target residual LAT risk in insulin-resistant populations. Prospective trials should clarify whether such interventions confer additive benefits beyond conventional anticoagulation in LAT prevention.

The integration of insulin resistance biomarkers (TyG index and TyG–BMI) into early risk stratification could revolutionize LAT prevention by enabling preemptive metabolic modulation. These indices, reflecting subclinical dysglycemia and lipid dysregulation, identify high-risk individuals before overt atrial structural or functional abnormalities emerge. Early intensification of therapies targeting hyperinsulinemia or hypertriglyceridemia may disrupt the “metabolic memory” driving chronic inflammation, fibrosis, and thrombogenic milieu (Zhong et al., 2024; Lui et al., 2023; O'Keefe et al., 2021; Reiner et al., 2024). For instance, SGLT2 inhibitors could attenuate sodium–hydrogen exchanger hyperactivity in atrial cardiomyocytes, thus reducing oxidative stress-linked calcium mishandling and stasis (Lui et al., 2023; O'Keefe et al., 2021). By intervening at the reversible stage of metabolic derangement—prior to the irreversible atrial substrate formation—such strategies might decouple insulin resistance from LAT pathogenesis, which is a critical advantage over late anticoagulation alone. While the current risk models lack granularity to capture this pre-thrombotic window, biomarker-guided early therapy could shift the paradigm from reactive thrombosis management to upstream metabolic prevention, potentially reducing anticoagulant dependence in selected populations. Validation in trials comparing early metabolic intervention versus standard care is imperative to quantify this transformative potential.

Despite the crucial findings being mentioned, our study has some limitations. First, this is a retrospective study with a relatively small sample size, and it is not specifically designed to assess the endpoints reported within this manuscript. Prospective studies are required to further confirm the results of our study. Second, other insulin resistance indicators, such as the homeostatic model assessment for insulin resistance, were not included due to the lack of clinical data. Furthermore, this study did not incorporate speckle tracking echocardiography (STE) to quantify left atrial strain and mechanical dysfunction—a potential precursor to thrombus formation (Providência et al., 2013; Kupczynska et al., 2017; Sonaglioni et al., 2021). The absence of STE data limits the exploration of whether impaired atrial contractility, driven by metabolic dysregulation, mediates the observed link between insulin resistance and LAT risk. Finally, although the sensitivity and specificity of TEE to diagnose thrombi with diameter ≥ 2 mm are nearly 100% and 99%, respectively, micro-thrombi (<2 mm) might be missed, especially in the left atrium appendage with a complex structure (Hahn et al., 2013).

## 5 Conclusion

In conclusion, elevated TyG index and TyG–BMI are associated with LAT in patients with NVAF. The TyG index and TyG–BMI may have the potential to be predictive biomarkers of LAT. Further studies are warranted to verify our findings.

## Data Availability

The data analyzed in this study is subject to the following licenses/restrictions: The datasets generated and analyzed during the current study are not publicly available due to the Henan Provincial People’s Hospital and Fuwai Central China Cardiovascular Hospital regulations, but are available from the corresponding author on reasonable request. Requests to access these datasets should be directed to Haixia Fu, fuxiami@163.com.

## References

[B1] Azarboo A. Behnoush A. H. Vaziri Z. Daneshvar M. S. Taghvaei A. Jalali A. (2024). Assessing the association between triglyceride-glucose index and atrial fibrillation: a systematic review and meta-analysis. Eur. J. Med. Res. 29 (1), 118. 10.1186/s40001-024-01716-8 38347644 PMC10860290

[B2] Bode D. Pronto J. R. D. Schiattarella G. G. Voigt N. (2024). Metabolic remodelling in atrial fibrillation: manifestations, mechanisms and clinical implications. Nat. Rev. Cardiol. 21 (10), 682–700. 10.1038/s41569-024-01038-6 38816507

[B3] Chan Y. H. Chang G. J. Lai Y. J. Chen W. J. Chang S. H. Hung L. M. (2019). Atrial fibrillation and its arrhythmogenesis associated with insulin resistance. Cardiovasc Diabetol. 18 (1), 125. 10.1186/s12933-019-0928-8 31558158 PMC6761716

[B4] Chen Y. Y. Liu Q. Liu L. Shu X. R. Su Z. Z. Zhang H. F. (2016). Effect of metabolic syndrome on risk stratification for left atrial or left atrial appendage thrombus formation in patients with nonvalvular atrial fibrillation. Chin. Med. J. Engl. 129 (20), 2395–2402. 10.4103/0366-6999.191744 27748329 PMC5072249

[B5] Cui C. Wu Z. Shi Y. Xu Z. Zhao B. Zhou D. (2021). Sex-specific association of BMI change with stroke in middle-aged and older adults with type 2 diabetes. Nutr. Metab. Cardiovasc Dis. 31 (11), 3095–3102. 10.1016/j.numecd.2021.07.007 34511289

[B6] Cui C. Qi Y. Song J. Shang X. Han T. Han N. (2024). Comparison of triglyceride glucose index and modified triglyceride glucose indices in prediction of cardiovascular diseases in middle aged and older Chinese adults. Cardiovasc Diabetol. 23 (1), 185. 10.1186/s12933-024-02278-z 38812015 PMC11138075

[B7] Er L. K. Wu S. Chou H. H. Hsu L. A. Teng M. S. Sun Y. C. (2016). Triglyceride glucose-body mass index is a simple and clinically useful surrogate marker for insulin resistance in nondiabetic individuals. PLoS One 11 (3), e0149731. 10.1371/journal.pone.0149731 26930652 PMC4773118

[B8] Gerrits A. J. Koekman C. A. van Haeften T. W. Akkerman J. W. (2010). Platelet tissue factor synthesis in type 2 diabetic patients is resistant to inhibition by insulin. Diabetes 59 (6), 1487–1495. 10.2337/db09-1008 20200314 PMC2874710

[B9] Gu H. Shao S. Liu J. Fan Z. Chen Y. Ni J. (2019). Age- and sex-associated impacts of body mass index on stroke type risk: a 27-Year prospective cohort study in a low-income population in China. Front. Neurol. 10, 456. 10.3389/fneur.2019.00456 31118920 PMC6504695

[B10] Guerrero-Romero F. Simental-Mendía L. E. González-Ortiz M. Martínez-Abundis E. Ramos-Zavala M. G. Hernández-González S. O. (2010). The product of triglycerides and glucose, a simple measure of insulin sensitivity. Comparison with the euglycemic-hyperinsulinemic clamp. J. Clin. Endocrinol. Metab. 95 (7), 3347–3351. 10.1210/jc.2010-0288 20484475

[B11] Hahn R. T. Abraham T. Adams M. S. Bruce C. J. Glas K. E. Lang R. M. (2013). Guidelines for performing a comprehensive transesophageal echocardiographic examination: recommendations from the American society of echocardiography and the society of cardiovascular anesthesiologists. J. Am. Soc. Echocardiogr. 26, 921–968. 10.1213/ANE.0000000000000016 23998692

[B12] Hajhosseiny R. Matthews G. K. Lip G. Y. (2015). Metabolic syndrome, atrial fibrillation, and stroke: tackling an emerging epidemic. Heart rhythm. 12 (11), 2332–2343. 10.1016/j.hrthm.2015.06.038 26142297

[B13] Hill M. A. Yang Y. Zhang L. Sun Z. Jia G. Parrish A. R. (2021). Insulin resistance, cardiovascular stiffening and cardiovascular disease. Metabolism 119, 154766. 10.1016/j.metabol.2021.154766 33766485

[B14] Hindricks G. Potpara T. Dagres N. Arbelo E. Bax J. J. Blomstrom-Lundqvist C. (2021). 2020 ESC guidelines for the diagnosis and management of atrial fibrillation developed in collaboration with the european association for cardio-thoracic surgery (EACTS): the task force for the diagnosis and management of atrial fibrillation of the european society of cardiology (ESC) developed with the special contribution of the european heart rhythm association (EHRA) of the ESC. Eur. Heart J. 42, 373–498. 10.1093/eurheartj/ehaa612 32860505

[B15] Jiang Y. Shen J. Chen P. Cai J. Zhao Y. Liang J. (2024). Association of triglyceride glucose index with stroke: from two large cohort studies and mendelian randomization analysis. Int. J. Surg. 110 (9), 5409–5416. 10.1097/JS9.0000000000001795 38896856 PMC11392123

[B16] Karam B. S. Chavez-Moreno A. Koh W. Akar J. G. Akar F. G. (2017). Oxidative stress and inflammation as central mediators of atrial fibrillation in obesity and diabetes. Cardiovasc Diabetol. 16 (1), 120. 10.1186/s12933-017-0604-9 28962617 PMC5622555

[B17] Kupczynska K. Michalski B. W. Miskowiec D. Kasprzak J. D. Wejner-Mik P. Wdowiak-Okrojek K. (2017). Association between left atrial function assessed by speckle-tracking echocardiography and the presence of left atrial appendage thrombus in patients with atrial fibrillation. Anatol. J. Cardiol. 18 (1), 15–22. 10.14744/AnatolJCardiol.2017.7613 28559531 PMC5512193

[B18] Lopez-Jaramillo P. Gomez-Arbelaez D. Martinez-Bello D. Abat M. E. M. Alhabib K. F. Avezum Á. (2023). Association of the triglyceride glucose index as a measure of insulin resistance with mortality and cardiovascular disease in populations from five continents (PURE study): a prospective cohort study. Lancet Healthy Longev. 4 (1), e23–e33. 10.1016/S2666-7568(22)00247-1 36521498

[B19] Lui D. T. W. Tang E. H. M. Wu T. Au I. C. H. Lee C. H. Woo Y. C. (2023). Risks of stroke, its subtypes and atrial fibrillation associated with glucagon-like peptide 1 receptor agonists versus sodium-glucose cotransporter 2 inhibitors: a real-world population-based cohort study in Hong Kong. Cardiovasc Diabetol. 22 (1), 40. 10.1186/s12933-023-01772-0 36829226 PMC9960638

[B20] Mancusi C. de Simone G. Best L. G. Wang W. Zhang Y. Roman M. J. (2019). Myocardial mechano-energetic efficiency and insulin resistance in non-diabetic members of the strong heart study cohort. Cardiovasc Diabetol. 18 (1), 56. 10.1186/s12933-019-0862-9 31039789 PMC6492323

[B21] Menke J. Lüthje L. Kastrup A. Larsen J. (2010). Thromboembolism in atrial fibrillation. Am. J. Cardiol. 105, 502–510. 10.1016/j.amjcard.2009.10.018 20152245

[B22] O'Keefe E. L. Sturgess J. E. O'Keefe J. H. Gupta S. Lavie C. J. (2021). Prevention and treatment of atrial fibrillation via risk factor modification. Am. J. Cardiol. 160, 46–52. 10.1016/j.amjcard.2021.08.042 34583808

[B23] Park J. W. Lee S. Y. Kim S. Y. Choe H. Jee S. H. (2008). BMI and stroke risk in Korean women. Obes. (Silver Spring) 16 (2), 396–401. 10.1038/oby.2007.67 18239650

[B24] Providência R. Faustino A. Ferreira M. J. Gonçalves L. Trigo J. Botelho A. (2013). Evaluation of left atrial deformation to predict left atrial stasis in patients with non-valvular atrial fibrillation - a pilot-study. Cardiovasc Ultrasound 11, 44. 10.1186/1476-7120-11-44 24354939 PMC3878330

[B25] Reiner M. F. Bertschi D. A. Werlen L. Wiencierz A. Aeschbacher S. Lee P. (2024). Omega-3 fatty acids and markers of thrombosis in patients with atrial fibrillation. Nutrients 16 (2), 178. 10.3390/nu16020178 38257071 PMC10821080

[B26] Shao Y. Hu H. Li Q. Cao C. Liu D. Han Y. (2024). Link between triglyceride-glucose-body mass index and future stroke risk in middle-aged and elderly Chinese: a nationwide prospective cohort study. Cardiovasc Diabetol. 23 (1), 81. 10.1186/s12933-024-02165-7 38402161 PMC10893757

[B27] Sonaglioni A. Lombardo M. Nicolosi G. L. Rigamonti E. Anzà C. (2021). Incremental diagnostic role of left atrial strain analysis in thrombotic risk assessment of nonvalvular atrial fibrillation patients planned for electrical cardioversion. Int. J. Cardiovasc Imaging 37 (5), 1539–1550. 10.1007/s10554-020-02127-6 33389359 PMC7778706

[B28] Tsai C. T. Chang S. H. Chang S. N. Hwang J. J. Wu C. K. Wang Y. C. (2014). Additive effect of the metabolic syndrome score to the conventional CHADS_2_ score for the thromboembolic risk stratification of patients with atrial fibrillation. Heart rhythm. 11 (3), 352–357. 10.1016/j.hrthm.2013.11.014 24252289

[B29] Zhan Y. Joza J. Al R. M. Barbosa R. S. Samuel M. Bernier M. (2018). Assessment and management of the left atrial appendage thrombus in patients with nonvalvular atrial fibrillation. Can. J. Cardiol. 34, 252–261. 10.1016/j.cjca.2017.12.008 29395705

[B30] Zhong J. Chen H. Liu Q. Zhou S. Liu Z. Xiao Y. (2024). GLP-1 receptor agonists and myocardial metabolism in atrial fibrillation. J. Pharm. Anal. 14 (5), 100917. 10.1016/j.jpha.2023.12.007 38799233 PMC11127228

